# Syphilis and HIV infections among pregnant women attending antenatal clinics in Republic of Congo

**DOI:** 10.11604/pamj.2017.28.8.13097

**Published:** 2017-09-07

**Authors:** Roch Fabien Niama, Nadia Claricelle Loukabou Bongolo, Edith Sophie Bayonne Kombo, Ruth Yengo, Pembe Issamou Mayengue, Etoka-Beka Mandingha Kosso, Igor Louzolo, Lucette Macosso, Ghislain Dzeret, Angélie Serge Patrick Dzabatou Babeaux, Marie-Francke Puruehnce, Henri Joseph Parra

**Affiliations:** 1 Laboratoire National de Santé Publique, 120 avenue du Général Charles de Gaule, BP: 120 Brazzaville, Congo; 2 Faculté des Sciences et Techniques, Université Marien Ngouabi, Brazzaville, République du Congo; 3 Faculté des Sciences de la Santé, Université Marien Ngouabi, Brazzaville, République du Congo; 4 Secrétariat Exécutif Permanent (SEP) du Conseil National de Lutte Contre le Sida: 2,459, Brazzaville, Congo; 5 Ministère de la Santé et de la Population, Programme National de Lutte Contre le Sida (PNLS), Brazzaville, Congo

**Keywords:** HIV, syphilis, pregnant women, Republic of Congo (RoC)

## Abstract

**Introduction:**

HIV and syphilis during pregnancy remain a public health concern especially in developing countries. Pregnant women attending antenatal clinics sites for the first time between September and December 2011 and who accepted to participate in the study were enrolled. The objective was to estimate the syphilis and HIV infection rate in this population.

**Methods:**

A study was conducted in 44 selected ANCs from 12 departments (5 urban and 7 rural). Pregnant women who accepted to participate in the study, attending selected sentinel ANCs sites for the first time between September and December 2011 were enrolled. To detect HIV antibodies, two consecutive ELISA assays were used (Genscreen Ultra HIV Ag/Ac, (BioRad, France) and Enzygnostic Intergral II (Siemens, GMBH, Marbug-Germany). In case of discordant results, the Western blot test II, HIV1 and 2 (Bio-Rad, Marne la Coquette, France) was used as the reference method. The RPR (Bio-Scan, Karnataka, India) test was performed to detect syphilis infection. The RPR positive results were confirmed using the TPHA test (Biotech, Cambridge, UK). Data were analyzed using SPSS 17.0 software.

**Results:**

A total of 2979 pregnant women attending ANCs were enrolled. The global HIV infection rate was estimated to be 3.6% (CI: 95%; 3.0-4.4). As expected, HIV prevalence was significantly higher in women aged above 25 years (4.4% (3.4-5.6), p = 0.026) and those attending urban ANCs (5.04%, p < 0.01). Also, women living in the urban area are more at risk to be infected (5.04 VS 2.38, p < 0.01). The RPR test was positive in 117 pregnant women (3.92%). The risk for syphilis occurrence was significantly higher among the single women compared to the married ones (4.4% VS 2.7%; p < 0.01). It was also estimated that the HIV and syphilis coinfection occurred in 22 cases (0.73%).

**Conclusion:**

The prevalence's of syphilis and HIV were relatively low. Marital status and sentinel site location were a risk factor associated with HIV and syphilis infections respectively. Therefore, substantial effort is needed to reinforce prevention strategies in this population to prevent mother-to-child and further horizontal transmissions of these infections.

## Introduction

Sexually transmitted infections (STIs) remain a public health problem in the world, particularly in developing countries [1,2]. In 2013, the number of people having STI was estimated around 1.3 billion [3]. Syphilis and HIV seem to be the most reported STIs. After experiencing a decrease tendency of syphilis prevalence, a recurrence cases is observed worldwide in the last decade (4). Indeed, the last estimation of WHO showed that approximately, 18 million of women worldwide are infected with syphilis and those with pregnancy have approximately 305 000 fetal and neonatal deaths every year and leave 215 000 infants at increased risk of dying from prematurity, low-birth-weight or congenital disease [3,4]. Infection with the Human Immunodeficiency Virus (HIV), responsible of the acquired immunodeficiency syndrome (AIDS) is also an important public health problem that affects many people around the world. The United Nations Program on HIV/AIDS (UNAIDS) estimated at 36.5 million, people living with HIV in 2014 [5]. Sub-Saharan Africa remains the most affected area with 70% of all cases [6]. In most developing countries particularly in Africa, epidemiological data on HIV and syphilis are often obtained from sentinel studies on pregnant women in antenatal consultation services in urban or rural areas. This is certainly due to funding constraints and difficulties related to logistics in the case of prevalence studies in the general population [7]. The Republic of Congo (RoC) is experiencing a HIV epidemic of a generalized type [8]. The seroprevalence survey conducted in 2009 in the overall population had allowed the collection of information needed to find the main indicators of HIV/AIDS in the country and a prevalence of 3.2% has been found [9]. However, significant interdepartmental disparities were observed with prevalence up to 4.8% in some departments. A tendency towards the feminization of the infection was observed (2.1% men vs 4.1% women). Regarding pregnant women, the latest available data from 2005 reported HIV prevalence to be 4.6% in this group [8]. However, no data on syphilis is available up to date. In order to define new control indicators against HIV and syphilis in this group, a global plan to fight neonatal HIV and syphilis transmission (plan eTME) was developed by the "Programme National de Lutte contre le Sida et les IST au Congo (PNLS)" in 2012. Among the measures suggested, it was decided to conduct a sentinel survey among pregnant women attending antenatal clinics to address the lack of data on STIs in this group. Therefore this study aimed to assess the magnitude of HIV and syphilis seroprevalence among antenatal clinics (ANCs) attendees in the RoC.

## Methods

A cross sectional study was conducted in the RoC, Central African country with an area of 342,000 km^2^. The RoC is divided into twelve administrative departments. At the last census conducted in 2007, the RoC's population was estimated at 3,697,490 inhabitants with a fertility rate of 2.8% [10].

**Site selection**: Taking into account the administrative subdivision of the country, a sentinel site was selected in each of the departmental capitals of the 12 departments. These sites were classified as rural or urban according to the official administrative subdivision. Therefore, five locations were classified as urban sentinel sites (Brazzaville, Pointe Noire, Nkayi, Dolisie, Ouesso) and seven others as rural sentinel sites (Sibiti, Madingo-Kayes, Owando, Ewo, Gamboma, Impfondo, Kinkala). In sentinel site selected health centers were considered, taking into account the possibility to provide antenatal clinic services, to prevent mother to children HIV transmission and the presence of an operational laboratories carrying out routine laboratory examinations including HIV testing and finally the possibility of keeping biological samples. A total of 44 ANCs were considered on which 24 and 20 were classified as urban and rural respectively.

**Data collection**: Between September and December 2011, all eligible pregnant women aged from 15 to 49 years old, who presented themselves for the first antenatal visit, were enrolled after informed consent was obtained. The following socio-demographic data were collected during individual interview using a structured questionnaire: age (subdivided from 20 per 5 years), marital status, education level, gravidity, parity and gestational age. A codified questionnaire completed by trained interviewers was used for data collection. An identification code had been edited to differentiate the sentinel site, the data collection center, the number of sample order. About 5 ml of whole blood were collected in EDTA tube. Plasma samples were then obtained after centrifugation for 10 minutes at 5000 rpm and then kept in cryovials in freezers (-20°C) before transfer to the Molecular Biology Unit of the National Laboratory of Public Health (NLPH) in Brazzaville, using a, ice-box.

**HIV and Syphilis detection**: In health centers, HIV infection was testing following the national HIV1/2 algorithm currently used in the country. Plasma samples were first tested using the Determine^®^ HIV1/2 (Alere Medical Ltd, Chiba, Japan) test. In case of positive result, the sample was subsequently tested with a discriminant rapid test, ImmunoComb^®^II HIV1/2 Bispot (Orgenics Ltd, P.O.B 360 Yavne 70650, Israel) used as a confirmatory test in the rural areas. If a confirmatory test was negative, the result was recorded as discordant. This discrepancy should be reported on the survey form for further investigation at the NLPH, where, after a quality control of samples (absence of hemolytic and samples well conserved), two other tests were carried out. In all samples, the ELISA Genscreen Ultra HIV Ag/Ac, (BioRad, France) was performed for the detection of IgG antibodies against HIV1/2. In case of negative result, the sample was considered as definitely negative. In case of positive result, a second ELISA Enzygnostic Intergral II (Siemens Health care Diagnostics Products, GMBH, Marbug-Germany) was used. In case of positive result to the second test, the sample was considered positive. In case of discordant results, the Western blot test II, HIV1 and 2 (Bio-Rad, Marne la Coquette, France) was used as the reference method. For syphilis investigation, all ANCs were trained to carry out the rapid plasma reagin test (RPR test). The following algorithm was applied: RPR test (Bio-Scan, Karnataka, India) was used as a screening test and positive samples were them analyzed in a second step with the TPHA test (Biotech, Cambridge, UK) as a confirmation test.

**Data processing**: All data were entered to Cs Pro version 4.0 software and then transferred to SPSS 17.0 software for statistical analysis. The confidence interval was set at 95% and the significance level at 5%. The test used for the comparison of observed values and the search for an association between HIV, syphilis and HIV/Syphilis coinfection by sentinel sites was the Kruskal-Wallis test. The logistic regression model was used to access the relationship between HIV and syphilis infections with selected sociodemographic variables. The multivariate binary logistic regression model was used to adjust the factors effect. The magnitude of associations was assessed using odds ratios with respective 95% CI. The tests with a P-value less than 0.05 were considered as statistically significant. The level of concordance between the results of HIV test from the sites and the NLPHwas evaluated with the Cohen's Kappa index (K) calculated using the formula K = (Po-Pe)/(1-Pe), where Po is the observed proportion and Pe the theoretical proportion. The K value was interpreted according to Landis and Koch scale for which K > 0.81 defines a very good accordance, K between 0.61 and 0.80, a good accordance and K between 0.41 and 0.60, an average accordance.

**Ethical considerations**: The study was conducted after obtaining clearance from the Comité d'Ethique de Recherche en Sciences de la Santé (CERSA) of the Ministry of Research and Innovation Technologies. During data collection, all pregnant women eligible to the study were asked to provide a signed informed consent before interview and blood collection. For pregnant women aged less than 18 years, the informed consent was obtained from parents. To do this, a statement summarizing the objectives of the investigation was read to each individual, in French or one of the two national languages (Lingala and Kituba). The interviews were conducted in private to ensure the confidentiality of the information collected.

## Results

In the total of 2979 samples recruited, 1712 (57.46%) pregnant women were from urban sentinel sites and 1267 (42.53%) from rural (Table 1). A total of 56 (1.85%) pregnant women were rejected to participate in the study.

**Table 1:** Number of samples collected by sentinel site

Sentinel site (department)	Area	Effective
Nkayi (Bouenza)	Urban	291
Brazzaville	Urban	486
Owando (Cuvette)	Rural	229
Ewo (Cuvette-Ouest)	Rural	154
Madingo-Kayes (Kouilou)	Rural	176
Sibiti (Lékoumou)	Rural	189
Impfondo (Likouala)	Rural	166
Dolisie (Niari)	Urban	219
Gamboma (Plateaux)	Rural	163
Pointe-Noire	Urban	459
Kinkala (Pool)	Rural	190
Ouesso (Sangha)	Urban	257
Total		2979

**Detection of HIV infection in sentinel sites and NLPH**: All samples collected from all 2979 pregnant women were tested for HIV at the ANCs as well as at the NLPH. All negative samples from the ANCs were confirmed to be negative at the NLPH. However, among 128 samples reported to be positive at the ANCs, only 108 were confirmed positive at the NLPH. Therefore, the observed and random unconformities between the two results were of 98 and 92%, respectively. The kappa coefficient was 0.81, corresponding to a very good agreement between the two results.

**Sociodemographic characteristic of study participants and risk factors for HIV infection**: The mean age of participants was 25.34-9.2 years old. The percentage of single pregnant women was higher (56.3%) compared to those married (33.4%) and widows and divorced women were a minority with only 6 and 5%, respectively. The proportion of pregnant women with middle school education level was 51.9% followed by those with primary school level (19.8%) and only 5.1% had higher education level. Approximately 78% of pregnant women reported being at their second gravidity and 47.9% reported having given birth at least once. Women aged less than 20 years old were the most represented group (29.6%). HIV prevalence was estimated at 3.6% (CI: 95%; (3.0-4.4)). However, when considered separately, significant disparities were found based on sentinel sites, (Table 2). The highest prevalence was observed in the sentinel sites of Pointe-Noire (6.8% (4.4-9.6)), followed by Niari (5.0% (5.5-8.8)) and Brazzaville (4.5% (2.9 - 6.9)), while the lowest prevalence were found in the sentinel sites of Pool (0.5% (0.0-2.9)) and Cuvette-Ouest (0.6% (0.0 -3.6)). To assess the influence of age on HIV positivity, the study population was divided into two groups with a cut-off of 25 years. The probability of having an HIV infection was significantly higher in the group with age more than 25 years old (4.4% (3.4 - 5.6) vs 2.2% (2.1-3.9), p = 0.026) with those between 35-39 years old having a greater risk of HIV infection (7.2%, Crude odds ratio (COR = 3.07 (95% CI: 1.64-5.74), p < 0.001) (Table 2. Pregnant women living in the urban areas were significantly more exposed to be infected with VIH (5.04% vs 2.38%; p < 0.001). No relationship was found between HIV infection and marital status, gravidity, parity as well as education level.

**Table 2:** Seroprevalence of HIV and multivariate analysis according to selected sociodemographic characteristics of study participants

Variables	Frequency	No reactive (%)	Reactive (%)	COR (CI 95%)	*P*-value	Adjusted OR (CI 95%)	*P*-value
**Age**							
< 20 years	882	859(97.4)	23(2.1)				
20-24 years	590	571(96.8)	19(3.5)	1.24 (0.67-2.30)	0.49	1.18 (0.63-2.20)	0.59
25-29 years	720	694(96.4)	26(3.5)	1.39 (0.79-2.47)	0.24	1.36 (0.76-2.44)	0.29
30-34 years	449	429(95.6)	20(4.2)	1.74 (0.94-3.20)	0.07	1.77 (0.95-3.31)	0.07
35-39 years	250	231(92.4)	19(7.2)	3.07 (1.64-5.74)	< 0.01	3.19 (1.68-6.06)	< 0.01
40 years or more	88	84	4(4.5)	--			
**Sentinel Site**							
Rural	1470	1435(97.6)	35(2.38)				
Urban	1509	1433(95.0)	76(5.04)	2.17 (1.45-3.27)	< 0.01	2.06 (1.35-3.14)	<0.01
**Marital status**							
Single	1 676	1615(96.3)	61(3.7)				
Maried	995	964(96.6)	31(3.4)	0.92 (0.60-1.41)	0.70	0.80 (0.49-1.19)	0.24
Divorced	5	5(100.0)	0(0.0)	--		--	
Widowed	6	5(83.3)	1(16.7)	5.21 (0.60-4.23)	0.40	5.37 (0.59-48.39)	0.13
Missing data	297	283	14	--			
**Education Level**							
Without education	276	265(96.0)	11(4.0)				
Primary School	589	576(97.8)	13(2.2)	0.54 (0.24-1.23)	0.14	0.62 (0.27-1.41)	0.25
Secondary school	1547	1486(96)	61(3.9)	1.04 (0.54-2.00)	0.90	1.07 (0.55-2.12)	0.84
High School	377	360(95.5)	17(4.5)	1.14 (0.52-2.47)	0.74	0.96 (0.44-2.12)	0.92
Higher education	152	146(96.1)	6(3.9)	0.99 (0.36-2.73)	0.98	0.86 (0.30-2.41)	0.77
Missing data	38	38	0	--	--	--	

**Sociodemographic characteristic of study participants and syphilis prevalence**: A total of 117 (3.92%) pregnant women were tested positive with RPR and confirmed by a TPHA test. The results of this infection based on sentinel sites are showed in the Table 3. The sentinel site Madingou-Kaye in Kouilou department was as the most affected (10.3%) while the sentinel site of Ewo has the lowest proportion. The risk of contracting syphilis was found to be significantly higher among married pregnant women than among single (95% CI, COR: 0.60 (CI: 0.38-0.94)), p < 0.007) (Table 3). The age group between 35 and 39 years is the most affected with syphilis (6%) while this infection rate appears low in the lowest age groups. No relationship was found between syphilis infection and gravidity as well as parity.

**Table 3:** Seroprevalence of syphilis and multivariate analysis according to selected sociodemographic characteristics of study participants, Congo.

Variables	frequency	No reactive n(%)	Reactive n(%)	COR (CI 95%)	*P*-value	Adjusted OR (CI 95%)	*P*-value
**Age**							
< 20 years	882	851(96.5)	31(3.5)				
20-24 years	590	573(97.1)	17(2.9)	0.81 (0.45-1.48)	0.50	0.84 (0.42-1.66)	0.62
25-29 years	720	685(95.1)	35(4.9)	1.40 (0.86-2.30)	0.17	1.44 (0.75-2.78)	0.27
30-34 years	449	433(96.4)	16(3.6)	1.01 (0.55-1.87)	0.96	1.01 (0.45-2.26)	0.98
35-39 years	250	235(94.0)	15(6.0)	1.75 (0.93-3.30)	0.08	1.82 (0.79-4.19)	0.15
40 years or more	88	85 (96.6)	3(3.4)	--	--	--	--
**Sentinel Sites**							
Rural	1470	1414(96.2)	56(3.8)				
Urban	1509	1448(96.0)	61(4.0)	1.06 (073-1.54)	0.74	0.99 (0.67-1.47)	0.97
**Marital status**							
Single	1 676	1602(95.6)	74(4.4)				
Maried	995	968(97.3)	27(2.7)	0.60 (0.38-0.94)	0.02	0.52 (0.33-0.84)	0.00
Divorced	5	4(80.0)	1(20.0)	5.41 (0.60-49.02)	0.13	6.81 (0.59-78.3)	0.12
Widowed	6	5(83.3)	1(16.7)	4.33 (0.50-37.5)	0.18	3.56 (0.39-32.6)	0.26
Missing data	297	283 (95.2)	14(4.7)	--	--	--	--
**Education Level**							
Without education	276	260(94.2)	16(5.8)				
Primary School	589	570(96.8)	19(3.2)	0.54 (0.27-1.07)	0.07	0.61 (0.30-1.22)	0.16
Secondary school	1547	1489(96.2)	58(3.8)	0.63 (0.36-1.11)	0.11	0.70 (0.39-1.27)	0.24
High School	377	362(96.0)	15(4.0)	0.67 (0.33-1.39)	0.28	0.81 (0.38-1.72)	0.58
Higher education	152	143(94.1)	9(5.9)	1.02 (0.44-2.37)	0.95	0.99 (0.40-2.47)	0.99
Missing data	38	38(100)	0 (0.0)	--	--	--	--

**Figure 1:**
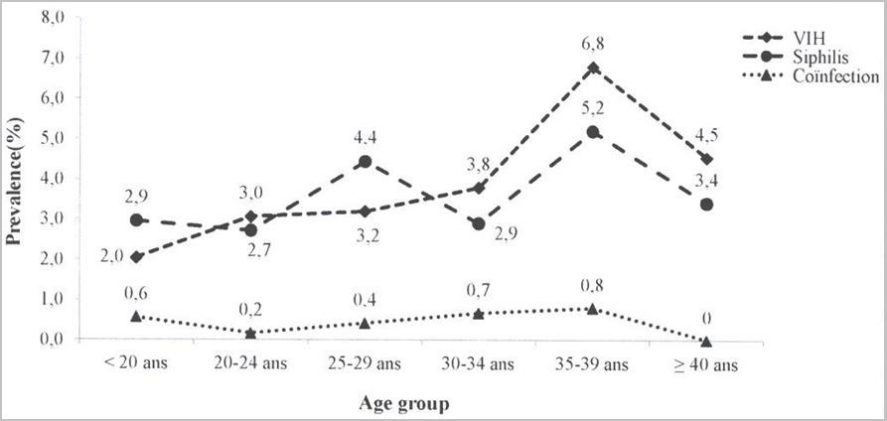
HIV, syphilis and coinfection prevalences among pregnant women according to age

**HIV and syphilis co-infection**: The association between HIV and syphilis does not seem very clear in this study. While in some departments, we observed comparable prevalence rates as in the case of the Sangha (3.1vs3.9 for HIV and syphilis, respectively), in the remaining sentinel sites, a certain dissociation seems to develop between the two infections. Likewise, a low rate of coinfection was observed (only 0.4%). However, it was observed that the highest HIV prevalence's were recorded in the sentinel sites where the prevalence of syphilis was also higher. This is the case of the sentinel sites of Pointe-Noire (HIV: 6.7% vs syphilis: 4.4%), Nkayi (3.4%vs10.3%) and Dolisie (5.02% vs 4.3%). The highest HIV and syphilis prevalence were observed in the sentinel sites of Pointe-Noire (6.7%) and Madingou-Kaye (10.3%), respectively. The highest rate of coinfection was however observed in the sentinel site of Impfondo (1.2%) as described in Table 4. Overall, the prevalence of HIV, syphilis and co-infection is increasing at any age, reaching a peak between 35 and 39 years as described in Figure 1.

**Table 4:** VIH and syphilis rate per sentinelles sites

Sentinels sites (department)	Area	Frequency	HIV Reactive (%)	syphilis reactive (%)	HIV and Syphilis reactive (%)	*P*-value
Nkayi (Bouenza)	Urbaine	291	10(3.4)	2(0.7)	0(0.0)	
Brazzaville	Urbaine	486	22(4.5)	14(2.9)	3(0.6)	
Owando (Cuvette)	Rurale	229	4(1.7)	10(4.4)	0(0.0)	
Ewo (Cuvette-Ouest)	Rurale	154	1(0.6)	1(0.6)	0(0.0)	
Madingo-Kayes (Kouilou)	Rurale	176	6(3.4)	18(10.3)	0(0.0)	0.20
Sibiti (Lékoumou)	Rurale	189	6(3.2)	3(2.0)	0(0.0)	
Impfondo (Likouala)	Rurale	166	4(2.4)	2(1.2)	2(1.2)	
Dolisie (Niari)	Urbaine	219	11(5.02)	11(4.3)	2(0.8)	
Gamboma (Plateaux)	Rurale	163	4(2.4)	7(4.3)	0(0.0)	
Pointe-Noire	Urbaine	459	31(6.7)	20(4.4)	4(0.9)	
Kinkala (Pool)	Rurale	190	1(0.5)	5(2.6)	(0.5)	
Ouesso (Sangha)	Urbaine	257	8(3.1)	10(3.9)	1(0.4)	

## Discussion

This study aimed to determine the prevalence of HIV and syphilis infections in pregnant women attending antenatal clinics in the RoC. HIV and syphilis infections were prevalent at 3.6% and 3.92%, respectively among pregnant women aged between 15 and 49 years old. Although the majority of study participants were single women (56.3%) living predominantly in urban cities (57.46%), the HIV prevalence observed in this study could be explained by the awareness of pregnant women about the risks of HIV infection following the effort of various fight against AIDS programs in the country, in raising awareness about the importance of screening and HIV prevention. HIV prevalence reported in this study is higher than that reported in some neighboring countries such as the Democratic Republic of Congo, where a prevalence of 1.9% was reported in rural and urban areas in 2011 [11] inversely lower compared to that reported in East-Africa were, in Angola, in the Luanda province, a prevalence of 8.8% was reported in 2015 [12] and in Tanzania, 5.6% of women was HIV infected in 2012 [13]. Other HIV seroprevalence studies were conducted in sub-Saharan Africa in the same population group, but restricted to hospital studies to urban or particular department in the country. This is the case of South-East Nigeria, where a prevalence of 5.1% was reported in 2010 in the hospital based study [14] and in the Mwandza department of Tanzania in 2006 where the prevalence of HIV was 7.6% [15]. This disparity in prevalence between the current study and those reported in other countries can certainly be explained by changes in risk sexual behavior and other risk factors that contribute to HIV transmission in this population. Interdepartmental disparities were significantly observed. The highest prevalence's were found in the southern departments of the country (Pointe-Noire, Niari, Kouilou, Bouenza and Lékoumou) except the Pool department. In the northern part of the country, all prevalence's were below the national average. This situation was also observed in 2009 in the general population survey [10]. The Reasons for this bipolarity is unknown. It is however believed that the high concentration of populations in the southern part of the country, as well as traditional practices such as scarification in rural healers, could explain this vulnerability [16,17]. Indeed, sociological studies are certainly needed to be carried out. Similar findings on the disparity in HIV prevalence among sites were observed in Cameroon between 2000 and 2006 [18].

According to the place of residence, a statistically significant difference was found between rural and urban areas regarding HIV infection. In fact, pregnant women living in urban area have a higher risk behavior. This difference was previously reported during the survey of HIV prevalence in the general population at national level in 2009 [8]. Similar findings have been reported in Ethiopia, showing a lower prevalence of HIV in rural areas in 2011 [19] as well as in Tanzania where the difference was most significant in 2015 [13]. In the Congolese context, this difference could be explained by a higher risk perception of HIV/AIDS considered as an extremely dangerous disease and pushing to more caution in rural, compared to relativism usually observed in urban areas, where AIDS is increasingly treated as a single chronic disease [10]. In a recent study in Tanzania, it was also shown that women in urban areas were significantly more vulnerable to HIV infection [20]. Current study, showed a significantly higher HIV prevalence among women more than 25 years old. The fact that older women have a longer sexual experience could have higher risk of HIV exposure. Similar results have been reported among women with age between 25 and 35 years old having more important levels of HIV infection in Tanzania and Ethiopia [13,17]. This risk is highly expressed for the age group 25-34 years as reported a study conducted by Manyahi et al [13] in Tanzania. No relationship was found for other sociodemographic factors such as education level and marital status. The overall prevalence of syphilis in the current study was 3.9%. A disparity between sentinel sites was also observed for this infection. Indeed, the greatest prevalence was reported in the south, particularly in the sentinel site Madingou-Kaye (10.3%) and in the city of Pointe-Noire (4.4%), the economic capital of the country, close to Madingou-Kaye. These high prevalence's may be related to the strong economic activity observed in the city of Pointe-Noire, increasing the risk behavior in particular infection in young unmarried women. Indeed, the analysis of risk for syphilitic infection has shown that only single pregnant women had a higher risk of contracting this infection compared to those married. Controversial findings have been reported is different studies conducted in the DRC, Tanzania and Ethiopia [11,13,19]. Many studies conducted in Sub-Saharan Africa show generally higher levels of prevalence. In neighboring countries of the RoC such as in the DRC and Angola, 4.2% and 8.8% of pregnant

## Conclusion

This study has presented the prevalence's of HIV and syphilis as well as their co-infection in pregnant women in the RoC. A disparity in prevalence has been reported between sentinel site and the most important being observed for these infections in the south of the country. Factors such as age greater than 25 years old and the fact of living in urban areas were positively associated with an increased HIV and syphilis risks behavior. However the intensification of prevention in this group might be necessary and help to further reduction of new infections in this group.

### What is known about this topic

- HIV and syphilis during pregnancy remains a public health concern especially in developing countries;
- STIs favor the occurrence of HIV infection;
- Very little data available regarding these diseases in Sub-Saharan Africa.

### What this study adds

- Better knowledge of syphilis infections and HIV among pregnant women in RoC;
- Disparity in prevalence based departments and sentinel surveillance type;
- Older age and marital statue are increasingly becoming an important risk factor.

## Competing interests

The authors declare no competing interests.
